# Study of the stress in adults diagnosed with meningioma: Insights from a tertiary neurosurgical hospital

**DOI:** 10.1002/cnr2.2105

**Published:** 2024-07-25

**Authors:** Karashash Menlibayeva, Chingiz Nurimanov, Saken Nuradilov, Serik Akshulakov

**Affiliations:** ^1^ Hospital Management Department National Centre for Neurosurgery Astana Kazakhstan; ^2^ Vascular and Functional Neurosurgery Department National Centre for Neurosurgery Astana Kazakhstan

**Keywords:** brain cancer, chronic stress, intracranial tumor, meningioma, perceived stress

## Abstract

**Background:**

Meningiomas are the most common type of primary brain tumor, originating from the meninges – the protective membranes that surround the brain and spinal cord. Several well‐studied risk factors for meningiomas include gender, age, radiation exposure, genetic factors, and hormonal factors. Moreover, the influence of a person's psycho‐emotional stateon their overall health and mental well‐being, specifically stress, iscurrently a significant and relevant topic of discussion.

**Aims:**

This case–control study aimed to study the association between perceived stress, chronic stress, and meningioma in adult patients.

**Methods and results:**

The study included cases, which comprised adult patients with histologically confirmed meningioma, and controls, consisting of adult patients with no history of brain cancer. Data collection involved the use of three types of questionnaires. The first questionnaire focused on patients' personal information, geographic factors, and lifestyle habits. Two additional questionnaires “The Perceived Stress Scale” and “The Chronic Stress Scale” were employed to assess perceived stress and chronic stress. The questioning was conducted by a neurologist. Microsoft Excel and Stata 14 were used for the data analysis. Overall, 148 questionnaires were completed and included in the analyses. The average age of participants was 45.60 ± 13.90 years. Females outnumbered males in both groups. Patients with meningioma diagnosis had a higher level of perceived high stress compared to those without meningioma (*p* = .045). Respondents without a diagnosis of meningioma have reported having more chronic stress in general and ambient problems (*p* = .004), financial issues (*p* = .006), work (*p* < .001), non‐employment (*p* = .008), love and marriage (*p* < .001), isolation (*p* < .001), and residence (*p* < .001). Patients with meningioma, however, had less chronic stress compared to meningioma‐free patients.

**Conclusion:**

This study revealed no discernible connection between stress and meningioma within our study sample. Further research with matched case–control methodology with a larger sample size is warranted to thoroughly evaluate the potential role of stress in patients with meningioma.

## 1 INTRODUCTION

Meningiomas are generally slow growing, usually noncancerous tumors that originate from the meninges, the protective membranes surrounding the brain and spinal cord. They are the most common type of primary brain tumor and account for approximately 40% of all primary brain and central nervous system tumors.^1^, ^2^, ^3^ The incidence of meningiomas is the highest among all brain tumors at 9.51 per 100 000 population, and their occurrence becomes more prevalent as patients age, with a higher frequency observed in women.^1^, ^4^

While most meningiomas are benign, some can be malignant or atypical, and their growth can occasionally lead to symptoms as they compress surrounding brain tissue.^5^ According to the recent World Health Organization (WHO) classification, meningioma is considered a single type with morphological characteristics represented by 15 different subtypes.^6^ Depending on the subtype, treatment options for meningiomas may include observation, surgery, radiation therapy, or in some cases, medication.^7^ The choice of treatment also depends on various factors, including the tumor's size, location, and the patient's overall health. Regular monitoring and medical evaluation are often necessary for patients diagnosed with meningiomas.

Although the exact causes of meningiomas are not fully understood, several risk factors have been strongly associated with an increased likelihood of developing meningioma. The well‐studied of them are gender,^8^ age, radiation exposure,^9^, ^10^ genetic,^11^, ^12^ and hormonal factors.^8^ In addition to these well‐established factors, the influence of a person's psycho‐emotional state on their overall health and mental well‐being is currently a prominent and pertinent subject of discussion, particularly with a specific focus on stress.

Hans Selye, a pioneering researcher in the field of stress, introduced the first and most generic concept of stress, defining it as a physiological and psychological response that occurs when a person perceives a discrepancy between the demands placed upon them and their ability to cope with those demands.^13^ In modern life, stress is described as a state of mental tension stemming from challenging situations. Stress represents an inherent human response that serves as a motivational force, propelling people to confront and effectively navigate the various challenges and adversities encountered in life.^14^

There are perceived stress and chronic stress, which are two different concepts related to how people experience and cope with stress. As reported by Cohen and colleagues, perceived stress is a person's evaluation of how stressful they find their current situation or circumstances.^15^ Chronic stress, in turn, is characterized by long‐term, ongoing stress that endures for an extended period. It typically results from prolonged exposure to stressors, such as continuous work‐related pressures, financial difficulties, relationship issues or recurring life challenges.^16^ The methodology for defining, assessing, and measuring chronic and perceived stress has not changed considerably since the late nineties. Recent studies continue to evaluate chronic stress by assessing major life domains, including the work environment, marriage, financial situation, and other chronic stressors, over a duration of at least six months. Acute, or perceived stress focuses on the peoples' subjective evaluation of stress in a specific situation.^17^ Compared to perceived stress, chronic stress can be challenging to manage because it may not have a clear endpoint, and people may adapt to a high‐stress baseline, making it their new, albeit unhealthy, normal. And the trend of people living in their “new normal” is increasing steadily, leading to stress being described as a “hidden epidemic”.^18^

Regardless of type, stress is blamed for causing various diseases, including cancer. It can have an indirect effect on tumor development by affecting the immune system,^19^ potentially weakening its ability to combat cancerous cells. Additionally, some studies have suggested that chronic stress may be associated with an increased risk of initiation and progression of cancer.^20^, ^21^ This association is believed to involve several cellular and molecular immunological factors that can be compromised in cases of chronic stress. The specific link between stress and brain cancer, however, remains unclear and studies on association of stress and meningiomas are limited to date. Given the existing gap in the literature and the widespread prevalence of stress in contemporary life, particularly alongside the high occurrence of meningioma in the population, the aim of this study was to examine the potential relationship between perceived stress, chronic stress, and the occurrence of meningioma in adult patients.

## 2 METHODS

### 2.1 Study design and participants

This observational case–control study was conducted at the neurosurgical hospital serving all the patients throughout the country. The cases involved patients treated for meningioma, all of whom underwent neurosurgical intervention at the Brain Surgery Department of the National Centre for Neurosurgery over a two‐year period spanning 2021 to 2022. Only those patients who underwent surgical intervention and agreed to participate in the study were interviewed, and their medical records were assessed. Controls were selected from the admission department of the hospital, comprising patients with no brain cancer but hospitalized with disc herniation. All the controls had an MRI verifying the absence of brain cancer. Patients' questionnaires were administrated by the neurologist.

### 2.2 Questionnaires and scoring

To collect the data, three types of questionnaires were used. The first questionnaire consisted of 18 questions intended to study patients' demographic, geographic characteristics, and features in nutritional and physical activities. The rest two questionnaires studied participants' perceived and chronic stress. To evaluate perceived stress, “The Perceived Stress Scale” assessment tool was used.^22^ The 10‐item tool asked participants about their feelings and thoughts during the last month. Patients indicated their answers by circling the variants from 0‐never to 4‐very often. The calculation of the Perceived Stress Scale was done by reversing some scores and summing according to the methodology provided by the developers. The scores range from 0 to 40 with the latter indicating a higher level of stress. Scores were categorized as low (0–13), moderate (14–26), and high (27–40).

Enduring or chronic stress was measured using “The Chronic Stress Scale” developed by Wheaton.^23^ Wheaton's stress measure is a 51‐item inventory of subjectively reported chronic stressors. The items were asked about the ongoing and enduring sources of stress in participants' life conditions and broken down into 13 subscales: general or ambient problems include items 1–3, financial issues (items 4–8), work (items 9–15), nonemployment (items 16 and 38), love and marriage (items 17–25), non‐relationship (items 26 and 27), divorce or separation (items 28 and 29), isolation (item 30), nonparent (item 31), parental/family (items 32–37), social life (items 39–42), residence (items 43–46), and health (items 47–51). The participants read each item and were asked to reply with not true (0), somewhat true (1), or very true (2). The scores for each subscale were calculated by summing and taking the average. In cases when the participant did not have a particular role, the item scored a zero.

The first questionnaire was in Russian and Kazakh languages. The remaining two were originally in English but had official translations into the Russian language, which were further translated into Kazakh for participants who preferred filling them out in Kazakh language.

### 2.3 Clinical characteristics of patients with meningioma

Medical records of participants diagnosed with meningioma were further examined to detail both patient and tumor characteristics. Tumor grading was performed according to the 2016 WHO classification, based on histological examination. Tumor size was determined using information from the preoperative MRI scan and the report provided by the radiologist. Tumor size was categorized as small (less than 3 centimeters), large (3–6 centimeters), and giant (more than six centimeters). Tumor location categorization was performed based on a previous study of meningioma^24^ and were divided into three main groups: convexity/falx/parasagittal, skull base tumors, and other. The skull base category encompassed areas such as the cavernous sinus, cerebellopontine angle, clinoid, clivus, foramen magnum, jugular foramen, middle fossa, olfactory groove, orbital, parasellar, petroclival, petrous, planum sphenoidale, posterior fossa, skull base, sphenoid wing, and tuberculum sellae. The “other” category included intraventricular and multifocal tumors that could not be easily classified into skull base, convexity, or falx locations.

### 2.4 Statistical analysis

Data was cleaned and coded using Microsoft Excel (Microsoft Office [Microsoft Corp., Redmond, Washington, USA]) and statistical analysis was performed on Stata 14 (StataCorp, College Station, Texas, USA). Descriptive statistics included reporting proportions, frequencies, and mean values ± standard deviation. Differences and relationships between variables were tested using Fisher's exact, chi2, t‐test, and Wilcoxon‐Mann Whitney where appropriate. Logistic regression model was built using a backward stepwise approach to identify factors associated with meningioma diagnosis. A Complete Case Analysis was applied for missing data in a regression analysis. A *p*‐value less than .05 was considered statistically significant.

## 3 RESULTS

Overall, 148 questionnaires were completed and included in the analyses. An average age of participants was 45.60 ± 13.90 years. Females outnumbered males in both groups. Overall, most of the participants were employed with 88.41% in control and 44.30% in case groups. The place of birth and live for the most of patients with meningioma was the north region of a country (27.85% and 32.91% respectively). Almost three quarters of patients with meningioma were married while this figure was 63.77% in a group with no meningioma. Merely 10% of patients with meningioma was smokers and 5% reported that they used to smoke, while among cancer‐free patients 23% have reported to be smoker and 16% used to smoke. Regarding the drink habits, the percentage of participants distributed equally between those who never drink an alcohol and those who drink at weekends and holidays (49.32% at each group). Within the groups, however, the proportion of alcohol consumption was twice as much in a group with no meningioma compared to meningioma group. A half of the participants do not drink coffee at all (50.68%), however almost 70% were theist with a habit to drink three and more cups a day. Regarding the energy drink consumption, the most of patients have reported that they do not drink power beverages (94.94% among cases and 75.36% among controls). One fifth of meningioma patients had a previous history of any cancer in close relative, while this figure was 43.48% among patients without diagnosis of meningioma. Almost a tenth (9.46%) of participants have reported to have diabetes, and one sixth (16.22%) had confirmed genetic disorders. Patients with no meningioma tend to be more physically active with 18.84% doing at least 3 times a week and more, and 37.68% doing less than 3 times a week. Patients with meningioma diagnosis had the higher level of perceived high stress in comparison with patients without meningioma (7.59% vs. 2.90%), the difference was statistically significant, albeit with a bordered *p*‐value of .05. Most of the participants had experienced negative events in a lifetime, both in case and control groups (63.29% and 60.87% respectively). Demographic and behavioral characteristics of the participants are given in Table 1.

**TABLE 1:** Demographic and behavioral characteristics of the participants.

Variable	Patients with meningioma (*n* = 79; 53.38%)	Patients with no meningioma (*n* = 69; 46.62%)	*p*‐value	All (*N* = 148; 100%)
Age (mean ± SD)	54.75 ± 11.54	35.13 ± 7.50	<.001	45.60 ± 13.90
Age group (years)			<.001	
18–30	2 (2.53)	23 (33.33)		25 (16.89)
31–50	20 (25.32)	44 (63.77)		64 (43.24)
51 and older	57 (72.15)	2 (2.90)		59 (39.86)
Sex			.004	
Female	70 (88.61)	48 (69.57)		118 (79.73)
Male	9 (11.39)	21 (30.43)		30 (20.27)
Employment status			<.001	
Employed	35 (44.30)	61 (88.41)		96 (64.86)
Unemployed	39 (49.37)	6 (8.70)		45 (30.41)
NA	5 (6.33)	2 (2.90)		7 (4.73)
Region of birth			<.001	
Central Kazakhstan	4 (5.06)	10 (14.49)		14 (9.46)
East Kazakhstan	14 (17.72)	1 (1.45)		15 (10.14)
North Kazakhstan	20 (25.32)	15 (21.74)		35 (23.65)
West Kazakhstan	14 (17.72)	22 (31.88)		36 (24.32)
South Kazakhstan	15 (18.99)	19 (27.54)		34 (22.97)
Other (outside Kazakhstan)	12 (15.19)	2 (2.90)		14 (9.46)
Region of childhood			.027	
Central Kazakhstan	6 (7.59)	7 (10.14)		13 (8.78)
East Kazakhstan	13 (16.46)	2 (2.90)		15 (10.14)
North Kazakhstan	22 (27.85)	15 (21.74)		37 (25.00)
West Kazakhstan	14 (17.72)	21 (30.43)		35 (23.65)
South Kazakhstan	17 (21.52)	21 (30.43)		38 (25.68)
Other (outside Kazakhstan)	7 (8.86)	3 (4.35)		10 (6.76)
Region of most lived			<.001	
Central Kazakhstan	6 (7.59)	15 (21.74)		21 (14.19)
East Kazakhstan	15 (18.99)	2 (2.90)		17 (11.49)
North Kazakhstan	26 (32.91)	9 (13.04)		35 (23.65)
West Kazakhstan	16 (20.25)	21 (30.43)		37 (25.00)
South Kazakhstan	16 (20.25)	22 (31.88)		38 (25.68)
Marital status			<.001	
Married	60 (75.95)	44 (63.77)		104 (70.27)
Single	2 (2.53)	19 (27.54)		21 (14.19)
Divorced	14 (17.72)	6 (8.70)		20 (13.51)
NA	3 (3.80)	0 (0.00)		3 (2.03)
Smoking			.005	
Yes	8 (10.13)	16 (23.19)		24 (16.22)
No, never	67 (84.81)	42 (60.87)		109 (73.65)
Used to smoke	4 (5.06)	11 (15.94)		15 (10.14)
Alcohol consumption			<.001	
No, never	51 (64.56)	22 (31.88)		73 (49.32)
Yes, at weekends and holidays	27 (34.18)	46 (66.67)		73 (49.32)
Yes, drink at least once a day	1 (1.27)	1 (1.45)		2 (1.35)
Coffee consumption			<.001	
1 cup a day	15 (18.99)	22 (31.88)		37 (25.00)
2–3 cups a day	3 (3.80)	21 (30.43)		24 (16.22)
4 and more cups a day	4 (5.06)	0 (0.00)		4 (2.70)
Do not drink	49 (62.03)	26 (37.68)		75 (50.68)
Occasionally	8 (10.13)	0 (0.00)		8 (5.41)
Tea consumption			.984	
1–2 cups a day	13 (16.46)	12 (17.39)		25 (16.89)
3 and more cups a day	54 (68.35)	47 (68.12)		101 (68.24)
Do not drink	12 (15.19)	10 (14.49)		22 (14.86)
Energy drink consumption			.001	
At least 1 a day	2 (2.53)	12 (17.39)		14 (9.46)
Occasionally	2 (2.53)	5 (7.25)		7 (4.73)
Do not drink	75 (94.94)	52 (75.36)		127 (85.81)
Previous history of any cancer in close relative (parents, siblings)			.002	
Yes	16 (20.25)	30 (43.48)		46 (31.08)
No	63 (79.75)	39 (56.52)		102 (68.92)
Genetic disorder			.418	
Yes	11 (13.92)	13 (18.84)		24 (16.22)
No	68 (86.08)	56 (81.16)		124 (83.78)
Diabetes			.014	
Yes, type 1	0 (0.00)	1 (1.45)		1 (0.68)
Yes, type 2	11 (13.92)	2 (2.90)		13 (8.78)
No	68 (86.08)	66 (95.65)		134 (90.54)
Physical activity			<.001	
At least 3 times a week and more	9 (11.39)	13 (18.84)		22 (14.86)
Less than 3 times a week	4 (5.06)	26 (37.68)		30 (20.27)
No	66 (83.54)	30 (43.48)		96 (64.86)
Perceived stress			.045	
Low stress	31 (39.24)	17 (24.64)		48 (32.43)
Moderate stress	42 (53.16)	50 (72.46)		92 (62.16)
High stress	6 (7.59)	2 (2.90)		8 (5.41)
Number of Experiences of negative events in a lifetime			.816	
No (0)	29 (36.71)	27 (39.13)		56 (37.84)
At least 1	20 (25.32)	20 (28.99)		40 (27.03)
2	16 (20.25)	10 (14.49)		26 (17.57)
3 and more	14 (17.72)	12 (17.39)		26 (17.57)

Table 2 presents the clinical characteristics of participants diagnosed with meningioma (*N* = 79). About a quarter of the patients (26.58%) had undergone previous surgery for meningioma. More than half of the patients presented with a Karnofsky Performance Status Scale of 70% upon admission to the hospital. The average time since the initial presenting symptoms was approximately 2 years, and since the radiographic diagnosis (MRI/CT) of the tumor, eight months. Most of the patients experienced frequent headaches (92.41%), followed by fatigue/ lethargy (58.23%) and dizziness/ shaky walking (40.51%). For 80% of patients, open microsurgery was performed, while the remaining patients underwent gamma knife surgery. The most common comorbidities included cardiovascular system diseases, such as arterial hypertension, congestive heart failure, coronary disease, and angina (59.49%), followed by the gastrointestinal system diseases. Nearly 60% of the tumors were WHO Grade I, with grades II and III each comprising about 20%. Most tumors were large (62.03%) and located in the skull base (46.84%).

**TABLE 2:** Clinical characteristics of meningioma patients.

Patients' Characteristic	Value, *n* (%)
Number of patients	79 (100)
Age (years, mean ± SD)	54.75 ± 11.54
Sex	
Female	70 (88.61)
Male	9 (11.39)
Disability	
Yes	5 (6.33)
No	74 (93.67)
Karnofsky Performance Status Scale	
60%	5 (6.33)
70%	45 (56.96)
80%	29 (36.71)
Time since initial presenting symptoms, (months, mean ± SD)	26.81 ± 37.43
Time since radiographic diagnosis (MRI/ CT) of meningioma (months, mean ± SD)	8.10 ± 17.79
Surgery	
Open surgery	62 (78.48)
Gamma Knife	17 (21.52)
Had a previous surgery on meningioma	
Yes	21 (26.58)
No	56 (73.42)
Initial presenting symptoms	
Frequent headache	73 (92.41)
Dizziness/ shaky walk	32 (40.51)
Fatigue/ lethargy	46 (58.23)
Visual impairment/ diplopia	20 (25.32)
Ahypnosia/	2 (2.53)
Sonitus/ diminished hearing	9 (11.39)
Nausea/vomiting	10 (12.66)
Seizures	11 (13.92)
Numbness in the limbs	11 (13.92)
Hypomnesia	13 (16.46)
Anosmia	4 (5.06)
Dysphasia	2 (2.53)
Prosopalgia/ facial numbness	6 (7.59)
Uncontrollable smile	1 (1.27)
Uroclepsia	1 (1.27)
Tremor	1 (1.27)
Photophobia	2 (2.53)
Comorbidities	
Cardiovascular System diseases (arterial hypertension, congestive heart failure, coronary disease, angina)	47 (59.49)
Gastrointestinal System diseases (chronic pancreatitis, chronic gastritis, chronic cholecystitis, peptic ulcer, hepatitis B and C)	34 (43.04)
Renal System diseases (chronic pyelonephritis, chronic renal disease, malignant neoplasm of the kidney, chronic glomerulonephritis, nephrolithiasis)	14 (17.42)
Respiratory System diseases (chronic bronchitis, pulmonary fibrosis)	25 (31.65)
Endocrine System diseases (Huntington's disease, multinodular goiter, immunologic thyroiditis, type 2 diabetes, cardiometabolic syndrome)	14 (17.72)
Ophthalmological Conditions (retinal angiopathy, cataract, astigmatic, manifest deviation)	31 (39.24)
Dermatological Conditions (chronic dermatitis)	1 (1.27)
Neurological System diseases (diabetic polyneuropathy)	1 (1.27)
Tumor Characteristics	
WHO grade	
Grade I	46 (58.23)
Grade II	17 (21.52)
Grade III	16 (20.25)
Size, diameters (cm)	
Small (>3)	16 (20.25)
Large (3–6)	49 (62.03)
Giant (<6)	14 (17.72)
Location	
Skull base	37 (46.84)
Anterior cranial fossa	11 (13.92)
Middle cranial fossa	12 (15.19)
Posterior cranial fossa	14 (17.72)
Convexity/falx/parasagittal	30 (37.97)
Falx/parasagittal	8 (10.13)
Convexity	22 (27.85)
Other	12 (15.19)

A multivariable logistic regression model was built to identify factors associated with the diagnosis of meningioma. Variables demonstrating statistical significance were included in the model (Table 3). Patients aged 51 years and older showed a markedly increased odds of meningioma diagnosis, being 31.59 times higher compared to their younger counterparts, after adjusting for other variables. Conversely, men demonstrated approximately 91% lower odds of being diagnosed with meningioma in comparison to women, even after adjusting for other variables (OR 0.09, CI 0.01 ± 0.36, *p* = .02). Furthermore, participants engaging in physical activity less than three times a week demonstrated significantly lower odds of being diagnosed with meningioma compared to those who were inactive.

**TABLE 3:** Bivariate and multivariate logistic results of factors associated with meningioma diagnosis.

Variable	COR (95% CI)	*p*‐value	AOR (95% CI)	*p*‐value
Age (years)				
18–30	1		1	
31–50	5.22 (1.12 ± 24.35)	.035	5.02 (1.77 ± 32.75)	.091
51 and older	32.75 (13.53 ± 46.92)	<.001	31.59 (26.9 ± 52.14)	.010
Sex				
Female	1		1	
Male	0.29 (0.12 ± 0.70)	.005	0.09 (0.01 ± 0.36)	.017
Employment status				
Unemployed	1		1	
Employed	0.09 (0.03 ± 0.23)	<.001	0.02 (0.01 ± 2.90)	.119
Marital status				
Single	1		1	
Married	12.95 (2.87 ± 58.53)	.001	2.12 (0.31 ± 14.32)	.438
Divorced	26.92 (4.78 ± 151.66)	<.001	1.98 (0.17 ± 23.41)	.586
Smoking				
No, never	1		1	
Yes	0.31 (0.12 ± 0.79)	.015	0.90 (0.12 ± 6.65)	.919
Used to smoke	0.23 (0.07 ± .076)	.016	1.85 (0.26 ± 13.29)	.539
Alcohol consumption				
No, never	1		1	
Yes, at weekends and holidays	0.25 (0.12 ± 0.50)	<.001	0.51 (0.14 ± 1.82)	.302
Yes, drink at least once a day	0.43 (0.03 ± 7.21)	.558	0.05 (0.01 ± 4.76)	.203
Previous history of any cancer in close relative (parents, siblings)				
No	1		1	
Yes	0.33 (0.16 ± 0.68)	.003	0.30 (0.07 ± 1.26)	.102
Physical activity				
No	1		1	
Less than 3 times a week	0.07 (0.02 ± 0.22)	<.001	0.05 (0.01 ± 0.42)	.005
At least 3 times a week and more	0.31 (0.12 ± 0.82)	.017	0.24 (0.03 ± 2.05)	.194
Perceived stress				
Low stress	1		1	
Moderate stress	1.46 (1.22 ± 1.95)	.035	1.72 (1.19 ± 2.72)	.630
High stress	1.64 (1.29 ± 9.06)	.047	1.89 (1.12 ± 2.33)	.134

There were statistically significant differences among patients with and without meningioma toward the perception of chronic stress. Respondents without diagnosis of meningioma have reported to have more stress in general and ambient problems, financial issues, work, non‐employment, love and marriage, isolation, and residence. Patients with meningioma, however, had less chronic stress compared to meningioma‐free patients. Mann–Whitney U‐test with post hoc Bonferroni correction with the significance level of 0.01 is given in Table 4.

**TABLE 4:** A Mann–Whitney U‐test for differences among patients with and without meningioma toward the perception of chronic stress.

Variables	Meningioma status (Y = 79, *N* = 69)	(Mean ± SD)	Sum of ranks	z‐value	*p*‐value
General or ambient problems	Yes	2.11 ± 1.69	5154.5	−2.857	.004
	No	2.86 ± 1.50	5871.5		
Financial issues	Yes	2.87 ± 2.74	5179.5	−2.739	.006
	No	3.96 ± 2.61	5846.5		
Work	Yes	3 ± 3.52	4842.5	−4.053	<.001
	No	5.04 ± 3.11	6183.5		
Non‐employment	Yes	0.61 ± 1.07	5262.5	−2.635	.008
	No	1.03 ± 1.18	5753.5		
Love and Marriage	Yes	2.03 ± 3.55	5019.5	−3.484	<.001
	No	3.71 ± 3.71	6006.5		
Non‐relationship	Yes	0.27 ± 0.70	4794	−5.059	<.001
	No	1.12 ± 1.28	6232		
Divorce or Separation	Yes	0.28 ± 0.66	5820	−0.374	.7
	No	0.26 ± 0.59	5206		
Isolation	Yes	0.27 ± 0.65	5166.5	−3.465	<.001
	No	0.57 ± 0.70	5859.6		
Non‐parent	Yes	0.11 ± 0.42	5656.5	−1.591	.1
	No	0.25 ± 0.60	5369.5		
Parental/Family	Yes	1.30 ± 2.55	5472.5	−1.774	.07
	No	1.46 ± 2.04	5553.5		
Social life	Yes	1.43 ± 1.58	5467.5	−1.656	.09
	No	1.83 ± 1.63	5558.5		
Residence	Yes	1.03 ± 1.54	5060.5	−3.370	<.001
	No	1.83 ± 1.72	5965.5		
Health	Yes	1.76 ± 1.94	6063	0.709	.4
	No	1.52 ± 1.91	4963		

## 4 DISCUSSION

This cross‐sectional study intended to study the potential association between perceived stress, chronic stress, and meningioma in adult patients. Although the study's results might not prompt noticeable responses, particular findings within the research hold the possibility of arousing academic curiosity.

Overall, about 70% of participants reported experiencing moderate to high levels of perceived stress, with meningioma group reporting slightly higher experience of high‐level perceived stress compared to non‐meningioma group (8% vs. 3%). This disparity may be indicative of the emotional and psychological challenges faced by people diagnosed with brain tumors. As reported by the scholars, a meningioma diagnosis can be a life‐altering event, leading to uncertainty, fear, and emotional distress for patients and their families.^25^, ^26^ Regardless of whether patients will have surgery or not, patients with meningioma might be under significant distress and may require psycho‐oncological support.^27^ The emotional burden caused by an upcoming surgery and being hospitalized in a brain tumor ward further contributes to the heightened perceived stress levels,^28^ which was observed in our study results. When discussing the post‐surgery mood of meningioma patients, it has been reported that the level of anxiety remains unchanged, while depression significantly decreases after the removal of the meningioma.^29^

The study revealed that patients with meningioma who reported experiencing two or more negative events throughout their lifetime had a marginally higher prevalence compared to those without brain cancer (38% vs. 32%). In our questionnaire, we asked respondents to provide details about the nature of these events, and most of their responses included the loss of close family members. It is worth noting that the repercussions of such a significant burden of loss are associated with an increased risk of developing multiple health disorders,^30^ including brain cancer. This observation aligns with the findings of a study conducted by Cabaniols et al, which established a connection between major life events and the risk of developing malignant primitive brain tumors.^31^

The findings from this study reveal intriguing insights into the relationship between chronic stress and the presence of meningioma. Notably, statistically significant differences were observed in the perception of chronic stress between participants. Meningioma free patients reported higher levels of chronic stress across various domains. These domains included general stress, ambient stressors, financial concerns, work‐related stress, non‐employment‐related stress, stress related to love and marriage, feelings of isolation, and stress associated with their place of residence. In contrast, patients diagnosed with meningioma demonstrated lower levels of chronic stress when compared to those without this type of brain tumor. This finding may raise questions regarding the potential protective or mitigating factors associated with a meningioma diagnosis.

Given that chronic stress is a persistent, long‐term condition, it is essential to consider such factors as age and gender, as there are demographic variations among the study participants. Older people often tend to perceive life stressors more readily and more attuned to various stressors in life than younger people due to their extensive life experiences and accumulated wisdom. It has also been reported the stress is experienced differently between men and women.^32^ These observations were evident in our study, which primarily involved older patients, with 89% of them being women in the meningioma group.

The consequences of chronic stress may vary and depends on individual coping mechanisms, social support networks, psychological resilience, and work and financial pressures, which were more pronounced in a group of meningioma‐free patients in our study. Most of them (88%) were employed and the findings regarding chronic stress could potentially be influenced by their employment status as stressors related to work can have a significant impact on mental health.^33^

Additionally, our research revealed a higher prevalence of chronic stress among patients without meningioma, which seemed related to relationship status. This is particularly noteworthy given the significant difference in the proportion of single people between those without meningioma (30%) and those with meningioma (3%), as well as the higher reported rates of occasional alcohol consumption among the former group (67% compared to 34%). These findings suggest that meningioma may develop independently of the presence of chronic stressors in patients.

**FIGURE 1:**
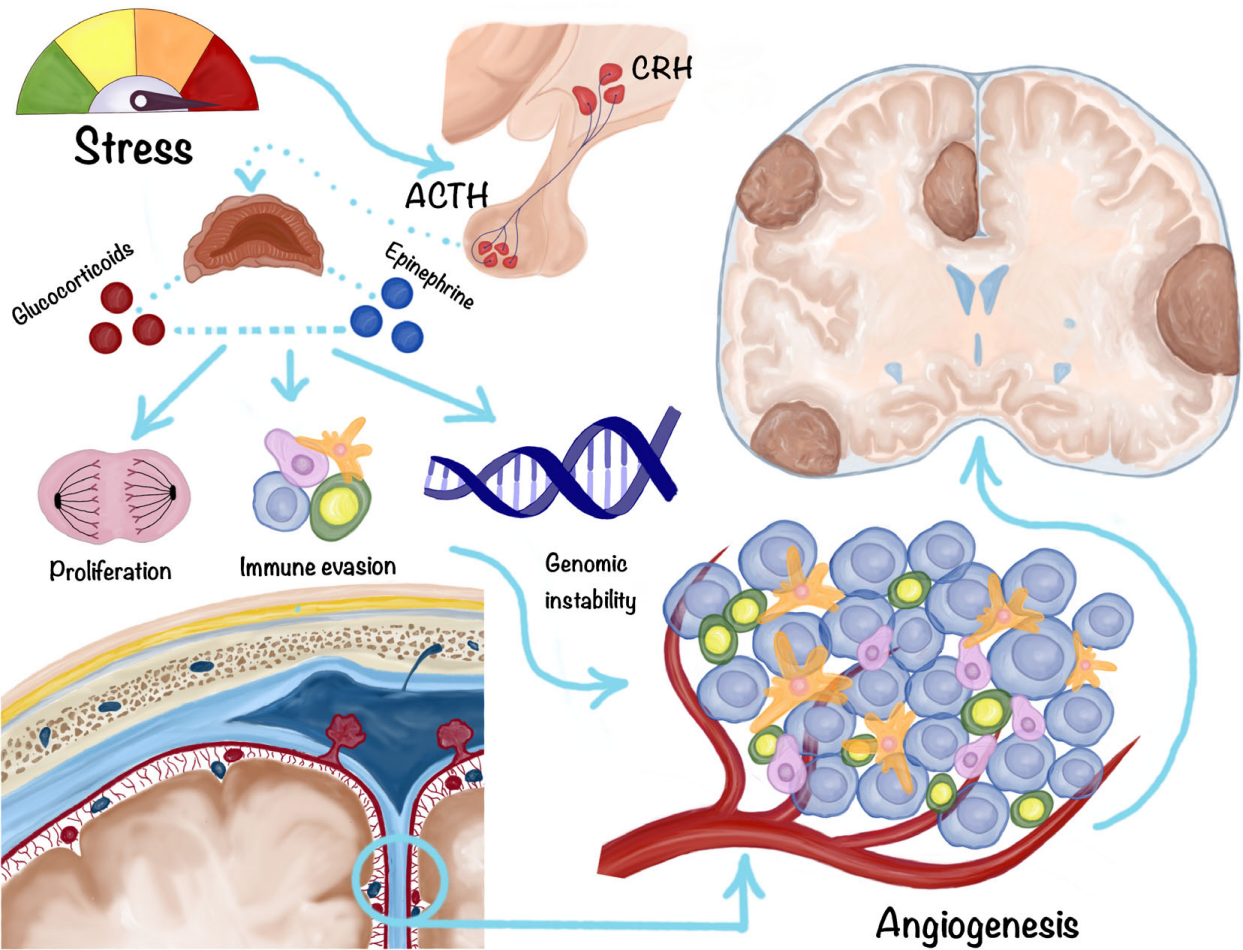
The potential role of stress in the development of brain tumors.

In case of malignant brain tumors, however, the findings suggest that chronic stress has the potential to disrupt various biological processes, promoting the development and progression of gliomas.^34^ The release of stress hormones, such as cortisol, can disrupt the normal functioning of the hypothalamic–pituitary–adrenal (HPA) axis, leading to dysregulation of stress response.^35^ Dysregulation of the HPA axis has been associated with increased inflammation, impaired immune function, and alterations in DNA repair mechanisms, which are all potential contributors to tumorigenesis.^36^ Furthermore, chronic stress can induce oxidative stress, causing an imbalance between reactive oxygen species and antioxidant defenses.^37^, ^38^ This imbalance can lead to DNA damage and mutations,^39^ facilitating the initiation and progression of tumors (Figure 1). Additionally, chronic stress has been linked to dysregulation of cellular signaling pathways, such as the mitogen‐activated protein kinase pathway,^40^ which plays a crucial role in cell growth and survival. Dysregulation of these pathways can promote uncontrolled cell proliferation and tumor formation.

While the evidence supporting this association is growing, further research is warranted to establish definitive causation and to explore effective therapeutic interventions. Understanding the intricate relationship between chronic stress and brain tumor development will have significant implications for both clinical practice and public health strategies. Further research is needed to elucidate the molecular mechanisms underlying this association and to identify specific stress‐related factors that may contribute to brain tumor initiation and progression.

### 4.1 Study limitations

This study has several noteworthy limitations that warrant consideration. The primary limitation is the use of an unmatched case–control design, which introduces the potential for selection bias. In this design, the cases and controls lack adequate matching in terms of age and gender, which introduces confounding variables and complicates the determination of whether the observed associations can be solely attributed to the exposure of interest. This study also suffers from self‐reported bias, which fail to provide more controlled evidence, demonstrating that chronic stress can accelerate brain cancer.

Furthermore, another limitation arises from the recruitment process, which focused solely on patients from a single national hospital while excluding participation from other regional hospitals. This limited scope restricts the study's applicability to the entire country, reducing the extent to which the findings can be applied to a more diverse and geographically varied population.

Thirdly, the control group comprised participants without brain cancer; however, there is no information available regarding whether the controls had other types of cancer aside from brain tumors, which could potentially impact stress levels. Furthermore, the inclusion of patients with disc herniation as controls raises additional concerns regarding the potential influence on the study results.

### 4.2 Clinical implications

Results of the study revealed an increased level of perceived stress among patients with meningiomas. Therefore, inpatient support of psychologists is essential for patients and their families.

### 4.3 Conclusion

In summary, our study did not find any significant association between stress and meningioma. It is important to note that the elevated levels of perceived stress observed among patients diagnosed with meningioma could likely be attributed to the actual diagnosis of meningioma. This is because the perceived stress scale measures stress levels in the past 30 days, a period during which patients were informed about their brain cancer diagnosis. Consequently, the heightened perception of stress among meningioma patients appears to be primarily linked to the diagnosis itself. Furthermore, our analysis revealed no discernible connection between chronic stress and meningioma within our study sample.

## AUTHOR CONTRIBUTIONS

**Karashash Menlibayeva:** Conceptualization (lead); data curation (equal); formal analysis (equal); investigation (lead); methodology (lead); writing – original draft (lead); writing – review and editing (lead). **Chingiz Nurimanov:** Data curation (equal); investigation (equal); writing – review and editing (equal). **Saken Nuradilov:** Data curation (equal); methodology (equal); writing – original draft (equal). **Serik Akshulakov:** Project administration (equal); supervision (equal); writing – review and editing (equal).

## CONFLICT OF INTEREST STATEMENT

The authors have stated explicitly that there are no conflicts of interest in connection with this article.

## ETHICS STATEMENT

The study was approved by the Institutional Review Board of the National Centre for Neurosurgery, protocol #3 dated 29 October 2021. Written informed consent forms were obtained from all participants.

## Data Availability

The data that support the findings of this study are available from the corresponding author upon reasonable request.
